# Cerebral Microbleeds and Their Association With Inflammation and Blood-Brain Barrier Leakage in Small Vessel Disease

**DOI:** 10.1161/STROKEAHA.124.048974

**Published:** 2025-01-02

**Authors:** Lupei Cai, Daniel J. Tozer, Hugh S. Markus

**Affiliations:** 1 Stroke Research Group, Department of Clinical Neurosciences, University of Cambridge, United Kingdom.

**Keywords:** blood-brain barrier, cerebral small vessel diseases, inflammation, microglia, positron emission tomography

## Abstract

**BACKGROUND:**

How cerebral microbleeds (CMBs) are formed, and how they cause tissue damage is not fully understood, but it has been suggested they are associated with inflammation, and they could also be related to increased blood-brain barrier (BBB) leakage. We investigated the relationship of CMBs with inflammation and BBB leakage in cerebral small vessel disease, and in particular, whether these 2 processes were increased in the vicinity of CMBs.

**METHODS:**

In 54 patients with sporadic cerebral small vessel disease presenting with lacunar stroke, we simultaneously assessed microglial activation using the positron emission tomography ligand [11C]PK11195 and BBB leakage using dynamic contrast enhanced magnetic resonance imaging, on a positron emission tomography–magnetic resonance imaging system. To assess local inflammation and BBB leakage, 3 one-voxel concentric shells were generated around each CMB on susceptibility-weighted imaging and resampled to positron emission tomography and T1 mapping images, respectively. In these 3 shells, we calculated the mean of PK11195 nondisplaceable binding potential (BPND) as a marker of microglial activation, as well as the mean influx rate as a marker of BBB leakage. In addition, 93 blood biomarkers related to cardiovascular disease, inflammation, and endothelial activation were measured to quantify systemic inflammation.

**RESULTS:**

No significant associations were found between the number of CMBs and the measures for microglial activation (*β*=2.6×10^−5^, *P*=0.050) and BBB leakage (*β*=−0.0001, *P*=0.400) in the white matter. There was no difference in measures of microglial activation (*P*=0.403) or BBB leakage (*P*=0.423) across the 3 shells surrounding the CMBs. Furthermore, after correcting for multiple comparisons, no associations were observed between systemic inflammation biomarkers and the number of CMBs.

**CONCLUSIONS:**

We found no evidence that CMBs are associated with either microglial activation assessed by [11]CPK11195 positron emission tomography or BBB leakage assessed by dynamic contrast enhanced magnetic resonance imaging, either globally or locally, in sporadic cerebral small vessel disease. There was also no association with markers of systemic inflammation.

Cerebral small vessel disease (cSVD) causes both lacunar stroke and intracerebral hemorrhage and is a major contributor to cognitive impairment and dementia.^1^ Cerebral microbleeds (CMBs) are one of the imaging hallmarks of cSVD.^2^ They are defined as small hypointense lesions on paramagnetic-sensitive magnetic resonance imaging (MRI) sequences and are thought to reflect small hemorrhages from disrupted cerebral small vessels.^3^ Owing to the paramagnetic properties of blood degradation products, CMBs can be detected by using gradient echo and susceptibility-weighted-imaging (SWI) MRI sequences. CMBs occur in a variety of different forms of cSVD including sporadic cSVD related to hypertension, genetic forms of cSVD, such as cerebral autosomal dominant arteriopathy with subcortical infarcts and leukoencephalopathy (CADASIL), and cerebral amyloid angiopathy (CAA). Their presence has been related to stroke recurrence^4^ and cognitive impairment independent of other MRI markers of cSVD.^5–7^ They may also reflect generalized microangiopathy presenting in other organs.^8^ However, how they arise and how they cause tissue damage are not fully understood. High blood pressure is most consistently reported as a risk factor for CMBs, in both stroke^9^ and population-based cohort.^10^ Other risk factors, including diabetes and hyperlipidemia, have been reported to be associated with CMBs in some but not all studies.^11^ It has been suggested that the formation of CMBs may be associated with increased blood-brain barrier (BBB) leakage,^12^ a feature noted in cSVD,^13^ and that they may be associated with inflammation which could exacerbate the tissue damage they cause.^14–16^

Inflammation is part of normal immune response to pathogens or endogenous stress signals. However, chronic inflammation due to dysregulated immune response has been linked to hypertension,^17^ diabetes, and cardiovascular diseases.^18^ More recently, evidence of both systemic and neuroinflammation has been found in cSVD through studies on inflammation biomarkers,^15^ neuropathology,^19^ and neuroimaging.^20^

Animal studies suggest that CMBs induce an inflammatory response. Cerebral microhemorrhages were induced with laser pulses in mice models. Migration and proliferation of microglia were observed adjacent to the acute CMBs within a radius of 200 µm with 2-photon excited fluorescence microscopy. This acute inflammatory response lasted for weeks.^21,22^ Neuropathological studies in man also support an inflammatory response. In CAA specimens, MRI-guided examination demonstrated microglial activation in the vicinity of CMBs.^23^ In vivo neuroinflammation can be imaged in humans using positron emission tomography (PET) with the TSPO (translocator protein) radioligands, such as PK11195. One previous study reported higher [^11^C]PK11195 ligand binding associated with both global and deep CMBs in patients with mild cognitive impairment or Alzheimer disease.^16^ CMBs have also been associated with systemic inflammation as estimated by inflammation biomarkers, including E-selectin, vascular endothelial growth factor, CRP (C-reactive protein), TNFR2, and myeloperoxidase.^15^

Increased BBB leakage has also been associated with CMBs. Increased CSF/serum albumin ratio, as a marker for BBB leakage, was associated with CMBs in a mixed cognitive impairment cohort.^24^ BBB leakage can also be measured in vivo in humans using the dynamic contrast-enhanced (DCE) MRI imaging method, and studies in monogenic cSVD and healthy individuals reported BBB leakage was related to CMB number.^25,26^

Previous studies in man have looked at associations between CMBs and markers of inflammation and BBB leakage across the whole brain. However, if CMBs are indeed associated with increased inflammation and BBB leakage this is likely to be in the local vicinity of CMBs. Therefore, we used DCE-MRI and [11C]PK11195 PET to determine whether there was evidence of microglial activation and BBB leakage in the local vicinity of the CMBs. We also explored the relationship between CMBs and systemic inflammation using 93 circulating biomarkers.

## Methods

The (anonymized) data that support the findings of this study are available from the corresponding author upon reasonable request by recognized researchers. This study follows the STROBE reporting guideline ([Strengthening the Reporting of Observational Studies in Epidemiology]; Supplemental Material).^27^

### Participants

Subjects were recruited with moderate to severe cSVD defined as symptomatic lacunar infarction in combination with MRI evidence of confluent white matter hyperintensities (WMH). The study involved a retrospective analysis of 54 subjects prospectively recruited as part of studies investigating both neuroinflammation and BBB leakage in cSVD.

**Figure 1.**
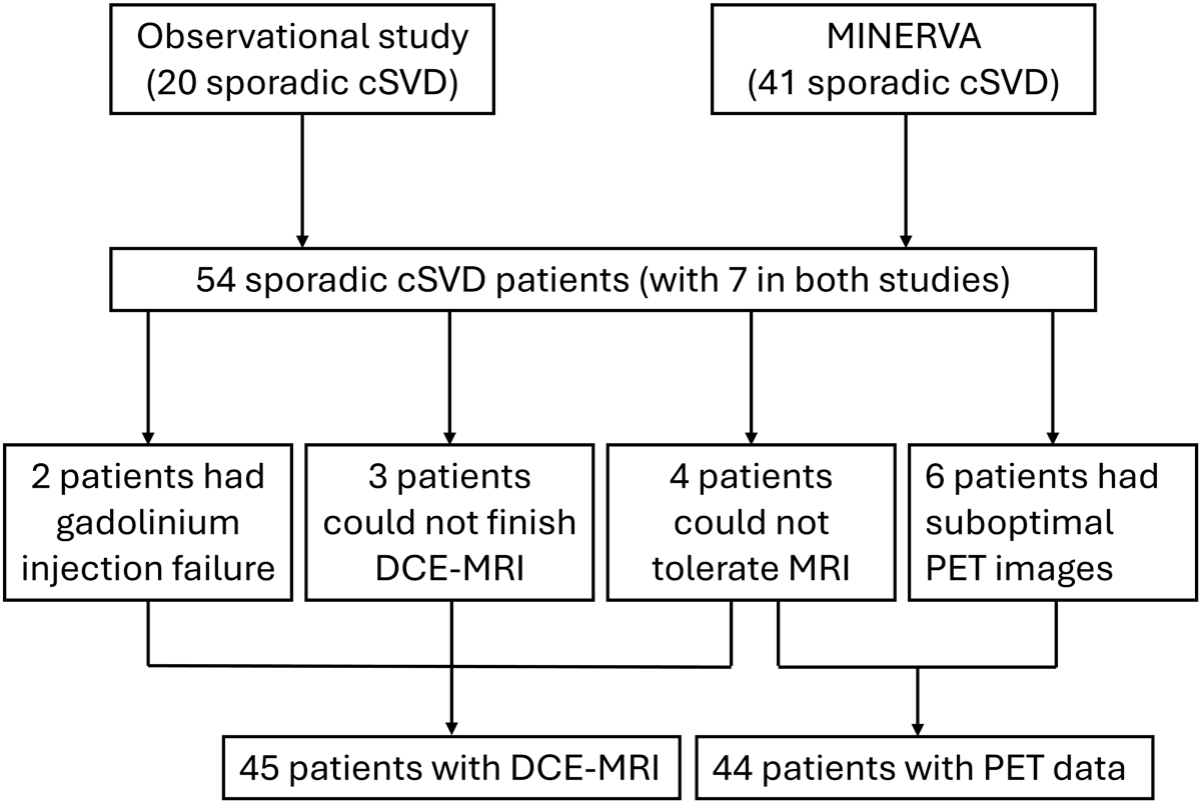
**Flowchart of patient inclusion.** cSVD indicates cerebral small vessel disease; DCE-MRI, dynamic contrast enhanced magnetic resonance imaging; MINERVA, Minocycline to Reduce Inflammation and Blood-Brain Barrier Leakage in Small Vessel Disease; and PET, positron emission tomography.

Patients were prospectively recruited from both inpatient and outpatient clinical stroke services at Cambridge University Hospital. Twenty patients participated in the initial observational study.^20^ An additional 34 patients participated in a follow-on randomized control trial, the MINERVA trial (Minocycline to Reduce Inflammation and Blood-Brain Barrier Leakage in Small Vessel Disease).^28^ The trial tested whether minocycline could influence novel pathological processes in SVD progression and showed no significant treatment effect. For all MINERVA trial participants, only the baseline data were used for this study. For the 7 patients who participated in both studies, only the data from the MINERVA trial were included. The detailed process is summarized in Figure 1.

The inclusion criteria for all participants were clinical evidence of lacunar stroke syndrome, with a corresponding lacunar infarct on diffusion-weighted imaging at the time of stroke for cases imaged within 3 weeks of stroke, or an anatomically compatible lacunar infarct (≤1.5 cm diameter) on fluid attenuated inversion recovery (FLAIR)/T1 MRI for cases imaged later after stroke. In addition, patients had to have confluent WMH defined as ≥2 on the Fazekas scale.^29^ Participants were not recruited until at least 3 months after a stroke to avoid transient changes in inflammation or BBB leakage secondary to the acute stroke.

Further exclusion criteria were any cause of stroke other than cSVD (eg, large artery stenosis >50% measured on NASCET criteria^30^ or a cardioembolic source) or a cortical infarct, contraindication to MRI, women of childbearing potential or who were breastfeeding due to the administration of the radioactive ligand, and an estimated glomerular filtration rate <60 mL/min per 1.73 m^2^ in view of the gadolinium contrast administration, unable or unwilling to consent, and dementia.

### Ethical and Regulatory Approval

Both the initial observational study and the MINERVA trial were approved by the East of England–Cambridge South Ethics Committee (REC no: 16/EE/0468, IRAS project ID: 212632), and the Administration of Radioactive Substances Advisory Committee (ref: 83/3886/35752). All participants provided written informed consent.

### Image Acquisition

All participants underwent noncontrast MRI, contrast-enhanced MRI, and PET in a single session on a GE SIGNA PET-MRI scanner (GE Healthcare) at the Wolfson Brain Imaging Center in Cambridge, United Kingdom.

PET and 3T MRI data were acquired simultaneously. A 32-channel NOVA head coil (Nova Medical) was used for MRI data acquisition. Noncontrast MRI sequences with the acquisition parameters included (1) isotropic 3-dimensional T1-weighted fast-spoiled gradient echo sequence (BRAVO), flip angle=12°, inversion time=450 ms, field of view=28 mm, slice thickness=1 mm, number of slices=192, reconstructed matrix size=512×512; (2) axial T2 fast-spoiled gradient echo sequence angled anterior commissure–posterior commissure, flip angle=111°, echo time=85 ms, repetition time=6000 ms, field of view=22 mm, slice thickness=5 mm, number of slices=31, reconstructed matrix size=1024×1024; (3) axial T2 FLAIR, angled anterior commissure–posterior commissure, flip angle=160°, echo time=120 ms, repetition time=8800 ms, inversion time=2445 ms, field of view=22 mm, slice thickness=5 mm, number of slices=28, reconstructed matrix size=256×256; (4) axial SWI, flip angle=17°, repetition time=40.6 ms, echo time=24.2 ms, field of view=22 mm, slice thickness=2 mm, number of slices=70, reconstructed matrix size=256×256; and (5) axial diffusion tensor imaging with angled anterior commissure–posterior commissure with the diffusion gradients applied in 63 directions with a *b* value=1000 s/mm^2^, TE=minimum, TR=15763 ms, field of view=19.2 mm, slice thickness=2 mm, number of slices=65–70 depending on slice angulation reconstructed matrix size=256×256.

Due to the limitation of temporal resolution, DCE-MRI could only be acquired in a subsection of the brain with characteristic cSVD imaging features. The subsection was chosen by a trained physician based on the noncontrast MRI before the DCE-MRI sequences. The scan started with precontrast T1 mapping followed by an injection of Gadoterate meglumine (Dotarem) at a dose of 0.025 mmol/kg and a rate of 6 mL/s. Follow-up T1 mappings were then performed for ≈25 minutes to map the changes in signal. The T1 mapping sequence used a 3-dimensional radiofrequency spoiled gradient echo imaging sequence with 6 flip angels (2°, 5°, 12°, 17°, 22°, and 27°). Eight postinjection maps with a temporal resolution of a total of 2.5 min were collected. The imaging parameters were echo time=1.784 ms, repetition time=6.3 ms, number of slices=16, reconstructed matrix size =256×256, and resolution=2×2×3 mm (reconstructed to=0.94×0.94×3 mm). In addition, a B0 mapping sequence for flip angle correction was acquired before contrast injection with the following parameters: flip angle=15°, number of echoes=1, receiver bandwidth=15.63, field of view=35 mm, and slice thickness=5 mm.

The [11C]PK11195 PET images were acquired following the injection of the [11C]PK11195 radiotracer. The trace was produced at the Wolfson Brain Imaging Center Radiopharmaceutical Unit and was injected over 30 seconds while list-mode PET data were acquired for 75 minutes. The median injected activity was 440 MBq (interquartile range, 401–483 MBq) with corresponding injected mass values of 3.9 µg (interquartile range, 2.8–6.4 µg).

### Noncontrast MRI Analysis

WMH volume was quantified using the semi-automated lesion contouring technique Jim analysis software (version 7.0.5, Xinapse Limited, http://www.xinapse.com) on FLAIR images by a single trained rater. Lacunes were defined as CSF-filled cavities at least 3 mm in diameter^^2^^ and were identified manually by a trained neurologist on FLAIR images, with both T1 and T2 images being inspected to exclude other lacune mimics. Diffusion data were analyzed with a diffusion tensor model at each voxel. The mean diffusivity in WM was measured as a conventional marker of diffusion tensor imaging metrics using histogram analysis as previously described.^28^

The FLAIR image was registered to the T1 image using a rigid body transformation in Advanced Normalization Tools (https://stnava.github.io/ANTs/). The resulting transformation was used to resample the WMH mask from the FLAIR image to the T1 image using nearest-neighbor interpolation. Each T1 image was processed using the segment routine in SPM12 (https://www.fil.ion.ucl.ac.uk/spm/software/spm12/). SPM segmentation provides tissue probability maps. The volumes for each tissue class were calculated as the sum of voxels that have a probability of >0.5 of belonging to that class, after removal of voxels in the WMH mask. The tissue segments and WMH mask were used to create the normal-appearing WM and WM (sum of normal-appearing WM and WMH) masks.

### DCE-MRI Analysis

DCE-MRI with fast T1 mapping and a low dose of gadolinium diethylenetriaminepentaacetic acid was used to quantitatively measure the BBB leakage.^31^ The influx rate (Ki) was determined by the Patlak graphical^32^ analysis as a metric of permeability on a voxel-by-voxel basis throughout the brain. To calculate Ki, it is necessary to determine the time course of the contrast agent concentration within arterial blood. As there is no artery in the field of view, the superior sagittal sinus was used as an arterial input function, corrected by the factor (1-hematocrit), which is assumed to be representative of the arterial concentration of the contrast agent. Mean Ki value was calculated in the WM, normal-appearing WM, and WMH masks to represent the BBB leakage of specific regions.

### PET Analysis

List-mode PET data were histogrammed into time bins and reconstructed into images (128×128×89 matrix; 2.0×2.0×2.8 mm voxel size) using time-of-flight ordered subsets expectation-maximization.^33^ Attenuation correction included the use of a multisubject atlas method^34^ and improvements to the MRI brain coil component. Image reconstruction was corrected for random coincidences, dead time, normalization, scattered coincidences, radioactive decay, and sensitivity.

SPM12 (https://www.fil.ion.ucl.ac.uk/spm/software/spm12/) was used to realign each dynamic image series. A mean realigned PET image was used to coregister each realigned dynamic PET image series to the T1 sequence from the same scan session.

The binding of the [11C]PK11195 was quantified with nondisplaceable binding potential (BPND), which was determined with a simplified reference tissue model incorporating correction for vascular binding correction.^35^ The WM reference tissue input was estimated with supervised cluster analysis^36^ using library data determined from healthy control [11C]PK11195 scans using the same PET-MRI scanner with the same acquisition and processing protocol.

### CMB Masks

CMBs were defined as small lesions, generally 2–5 mm and up to 10 mm in diameter, of low signal on susceptibility-weighted MRI images.^2^ They were manually marked and counted on SWI images by a single trained rater based on the BOMBS criteria.^37^ The CMB masks were acquired using the semi-automated lesion contouring technique Jim analysis software (version 7.0.5, Xinapse Limited, http://www.xinapse.com) on SWI image by the same rater.

SWI images underwent N4 bias correction^38^ before registration. The image was first registered to T1-weighted image and then to a T1 mapping image and PET image separately by rigid body registration using Advanced Normalization Tools (http://stnava.github.io/ANTs/). The resulting transformations were used to resample the CMB masks from the SWI space to the T1 mapping space and to the PET imaging space independently using nearest-neighbor interpolation.

### CMB Penumbras

**Figure 2.**
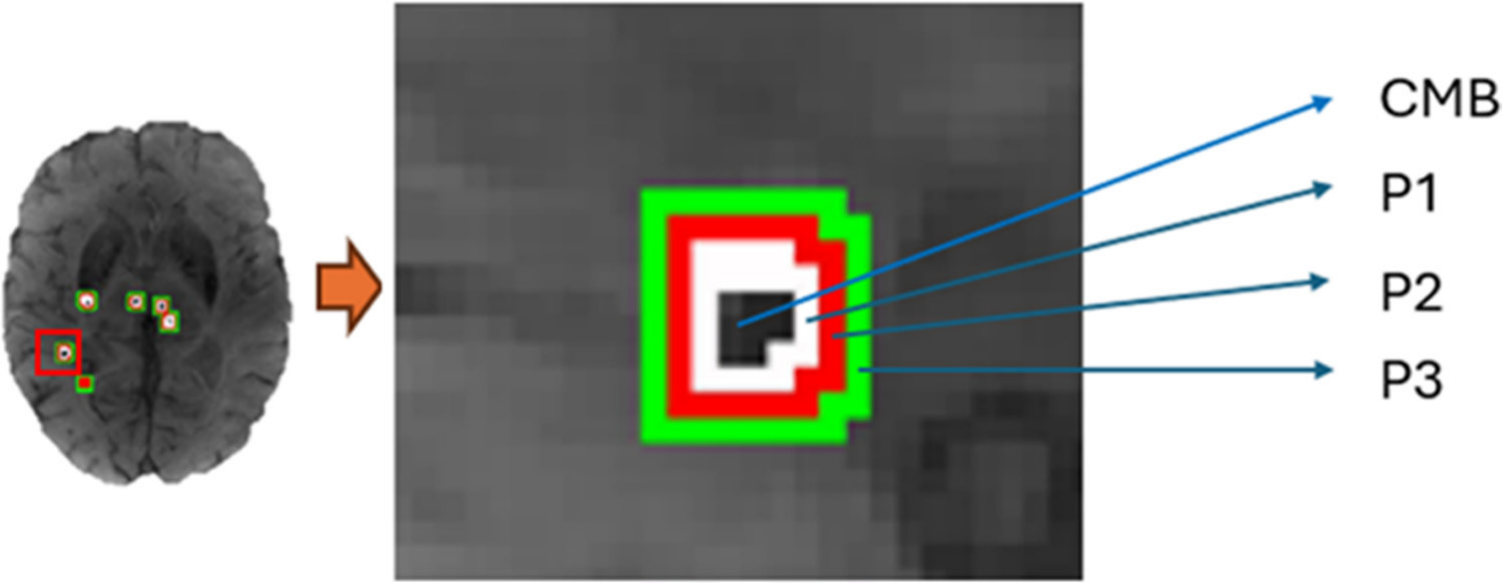
Close-up of a cerebral microbleed (CMB) with 3 one-voxel concentric shells comprising the CMB penumbra (P); P1–3 shows penumbra progressively further away from the CMB.

To assess the spatial associations between CMB and both BBB leakage and [11C]PK11195 ligand binding, a concentric series of penumbras around the CMBs was generated. CMB masks in T1 mapping space were dilated by 1 voxel in all directions; this was performed 3 times using FSLMaths (https://fsl.fmrib.ox.ac.uk/fsl/fslwiki) to derive 3 concentric shells. The penumbra masks were then overlaid onto the WM masks to retain only the voxels within the WM region. The same process was repeated for CMB masks in PET imaging space. The postprocessing image is shown in Figure 2. The BPND from the PET imaging data and the Ki from the DCE-MRI data were obtained for each final penumbra mask for analysis.

### Blood Biomarkers

Blood was taken on the same day as the PET-MRI scan for each participant. The blood was centrifuged, and the serum was separated and stored at −70°C. A panel of 93 blood biomarkers related to cardiovascular disease, inflammation, and endothelial activation were measured in each participant. This comprised 92 biomarkers from the Olink Proteomics Cardiovascular Disease III panel (https://www.olink.com/products/olink-target-96), together with CRP measured using the Siemens Dimension high sensitivity CRP assay at the Core Biochemical Assay Laboratory, Addenbrooke’s Hospital, Cambridge, United Kingdom. All assays were performed at the same time to avoid batch effects and were conducted blinded to subject identity.

### Statistical Analysis

All the statistical analyses were performed in R studio (version 4.3.3 2024, R Foundation For Statistical Computing, Vienna, Austria, https://www.r-project.org/).

First, we analyzed whether the presence of CMBs was associated with higher WM Ki and [11C]PK11195 BPND. The presence or absence of CMB was considered a binary variable. The mean Ki and BPND values obtained with the WM mask were used as markers for BBB leakage and microglia activation and were considered dependent variables in this analysis. They first underwent cube root transformation for normalization. A 2-sample *t* test was then used to compare the 2 groups.

After that, a linear regression model was used to analyze associations between mean Ki and BPND in the WM and total CMB number. Age and sex were included as covariates in the primary analysis, and the results were further adjusted for WMH volume, lacune number, and mean diffusivity peak height in the sensitivity analysis.

To analyze spatial association, mean Ki and BPND values in the 3 CMB penumbras were first cube root transformed for normalization. An ANOVA test was used to assess the relationship among the mean Ki and BPND values in the 3 penumbras, respectively. A post hoc Tukey test was then conducted to compare the difference between each pair of penumbras.

To assess associations between blood biomarkers and CMB number, all 93 biomarkers first underwent Shapiro-Wilk test for normality check. All non-normally distributed biomarkers were subjected to either a logarithmic or cube root transformation before any analysis. A linear regression model was adopted to analyze the association between the number of CMBs and individual biomarkers with analyses controlled for age and sex. Results were corrected for multiple comparisons using Benjamin-Hochberg method with a false discovery rate threshold of 5%. We also used principal component analysis to summarize the variation of the 93 biomarkers. This is to reduce the dimensions of the biomarker dataset to minimize multiple comparisons. The first 3 principal components were obtained based on the visual elbow method from the scree plot (Figure S1) for further analyses. Partial correlation analysis was then employed to investigate the association between the CMB number and individual principal component, controlling for age and sex.

## Results

### Cohort Details

Among the 54 patients included, 4 could not tolerate the PET-MRI scan, 3 did not complete the DCE-MRI sequences, 2 had gadolinium injection failure, and 6 had suboptimal PET image quality. Therefore, DCE-MRI data were available for analysis in 45 patients and PET data in 44 patients. The detailed cohort flow chart is depicted in Figure 1. The distribution of demographics, vascular risk factors, and cSVD imaging markers are summarized in Table S1.

### Association of CMB With BBB Permeability and Neuroinflammation

There was no significant difference of the mean Ki (*P*=0.585) and [11C]PK11195 BPND (*P*=0.425) in the white matter between the groups with CMB and without and no association between either parameter and CMB number (Tables 1 and 2).

**Table 1.**
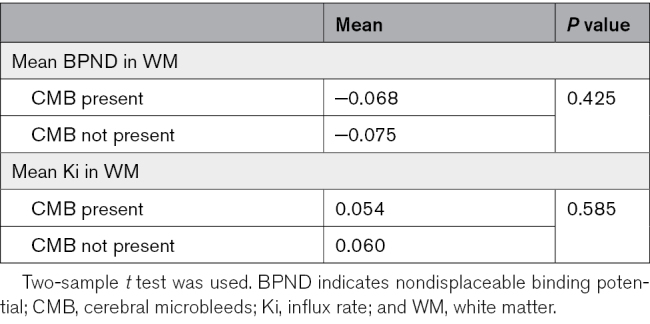
The Difference of BPND and Ki Between Group With CMB and Group Without CMB

**Table 2.**
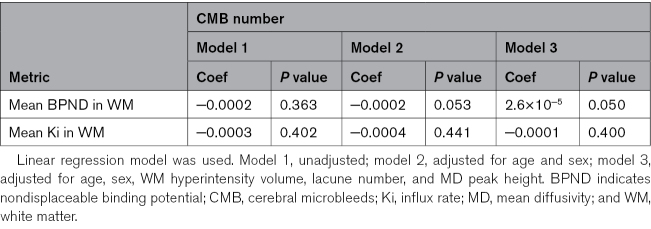
The Association Between CMB Number and Mean BPND and Ki in WM

**Figure 3.**
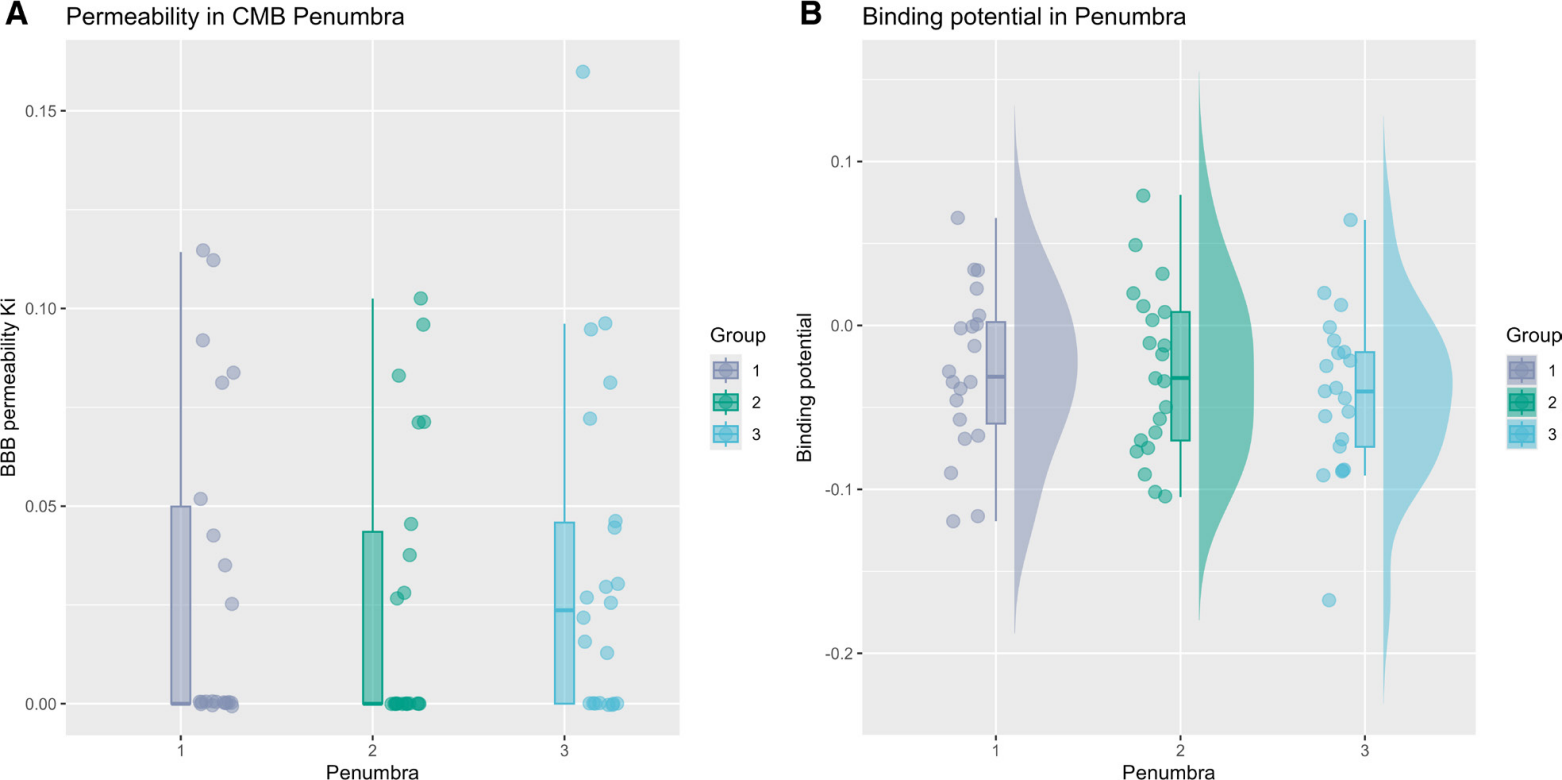
**Spatial analysis of blood-brain barrier (BBB) leakage (influx rate [Ki]) and [11C]PK11195 binding potential (BPND) in the vicinity of cerebral microbleeds (CMB). A**, Comparison of mean Ki in CMB penumbras. **B**, Comparison of mean BPND in penumbras.

We then studied whether the regions closer to the CMB have higher Ki and [11C]PK11195 BPND. No significant differences were observed in the mean Ki (*P*=0.423) or BPND (*P*=0.403) in the 3 penumbras around the CMB (Figure 3), demonstrating no evidence for local inflammation or increased BBB leakage in the vicinity of CMB.

### Association of CMB With Blood Makers of Inflammation

We next investigated whether the higher CMB number is associated with specific blood biomarkers. Seven (U-PAR, PCSK9, CPA1, CD163, CCL15, CASP-3, and BLM hydrolase) of the 93 biomarkers were significantly associated with the number of CMB after controlling for age and sex. However, none remained significant after false discovery rate correction (Table S2). In the principal component analysis, the first 3 components explained most of the variance and were used to represent the biomarker data (Figure S1). No association was found between the CMB number and any of the 3 principal components (Table S3).

### Association of New CMB With BBB Permeability and Neuroinflammation

**Figure 4.**
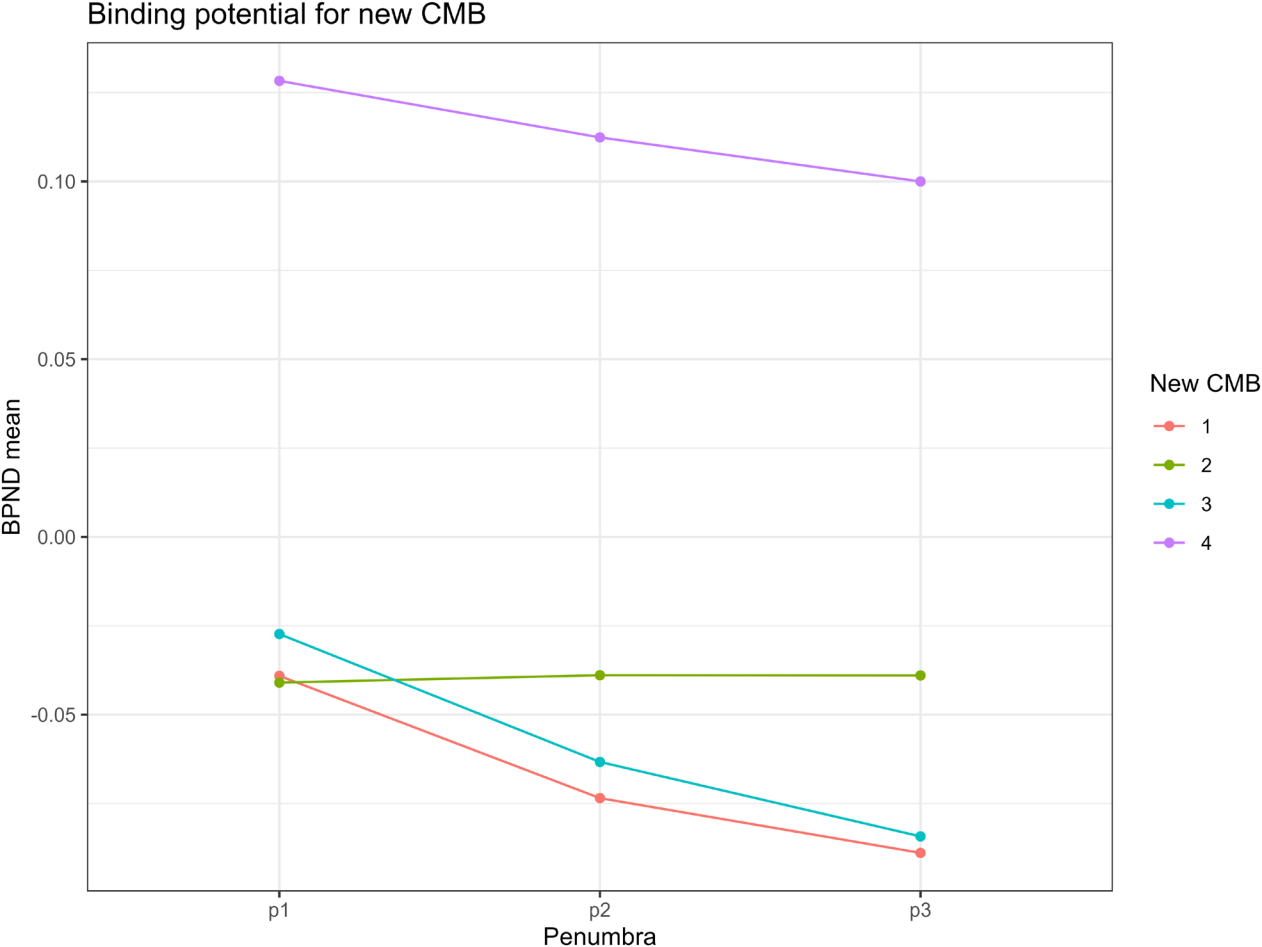
Mean 11C-PK11195 binding potential (BPND) in the penumbras of 4 new cerebral microbleeds (CMB).

It is possible neuroinflammation and BBB leakage might only be associated with CMB for a limited time period after their formation. To explore this, we identified 7 patients who were in both the initial observational study and the MINERVA trial and, therefore, underwent PET-MRI scans at 2 time points, with an average time interval of 29.3 months. Among them, 4 new CMBs were identified, and we assessed parameters in the vicinity of these new CMBs using the same method. We observed a trend towards PK11195 BPND and the distance to CMB, as shown in Figure 4, although this trend did not reach statistical significance. No similar trend was observed for BBB leakage.

## Discussion

In this study, we did not find evidence that CMBs are associated with either BBB leakage assessed by DCE-MRI or microglial activation assessed by [11C]PK11195 PET, either globally or locally, in sporadic cSVD. In addition, there was no association between the CMBs and biomarkers of systemic inflammation.

It has been suggested that CMBs are accompanied by a local inflammatory response, and it is possible this could increase the damage they cause and, therefore, the consequences of cognition. Support for this comes from both animal studies as described in the introduction, and a previous study reported higher [11C]PK11195 ligand binding associated with both global and deep CMBs in patients with mild cognitive impairment or Alzheimer disease.^16^ However, whether this association is causal or merely due to CMBs being a marker for cSVD which itself has been associated with increased inflammation^20^ is uncertain. In particular, whether CMBs are associated with a local inflammatory response has not been examined in humans. To address this, we determined [11C]PK11195 ligand binding in the vicinity of CMBs and assessed whether it reduced as one moved further away from the CMBs. We found no evidence of a local inflammatory response. We also found no association between CMB burden and global [11C]PK11195 ligand binding, or with circulating inflammatory markers. Therefore, our results do not support inflammation being associated with CMBs. This result differs from the study on patients with mild cognitive impairment or Alzheimer disease mentioned above.^16^ The differences in study cohorts may contribute to this discrepancy, as we specifically focused on sporadic cSVD and excluded cases with dementia or CAA.

One of the challenges in studying CMBs is the difficulty in accurately determining their time from onset. It is possible that some CMBs have been present for several years. This means that we could miss a transient inflammatory response occurring at CMB onset, which has resolved by the time we performed imaging. To address this, we explored data from the 7 patients who underwent [11C]PK11195 PET scans at 2 time points, with an average gap of 29.3 months. Among them, 4 new CMBs were identified, and we found a trend of an inverse association between PK11195 BPND and the distance from the CMBs. This would be consistent with a transient inflammatory response but is hypothesis-generating due to the small sample size.

Another feature described in cSVD is increased BBB leakage.^39–41^ Histopathology studies revealed that the majority of cortical CMBs originate from penetrating cortical arterioles in patients with CAA.^42^ The underlying vasculopathy of deep CMBs in hypertensive cSVD is less clear. However, hypertensive cSVD mainly affects arterioles with diameters between 40 and 900 µm.^8^ It is possible that deep CMBs originate from vessels of similar size. There is BBB breakdown at the site of a ruptured cerebral blood vessel, hence it is reasonable to hypothesize that the CMBs should be associated with BBB leakage. However, we did not observe any relationship between CMBs and increased BBB leakage, either globally or in the vicinity of CMBs. Therefore, we found no evidence supporting the role of increased BBB leakage in CMB formation. However, we cannot exclude that there is a transient increase of BBB leakage at the time of formation of CMBs, which had returned to normal by the time we performed imaging. The result is consistent with a recent imaging study, in which they found no association between CMBs and BBB leakage in 30 patients with sporadic cSVD. In addition, they demonstrated different patterns of BBB dysfunction among different subtypes of cSVD.^25^ This may explain the positive finding of CMBs and BBB leakage in cohorts with mixed cognitive impairment and CAA.^24,43^

Seven (U-PAR, PCSK9, CPA1, CD163, CCL15, CASP-3, and BLM hydrolase) of the 93 biomarkers were significantly associated with the number of CMB after controlling for age and sex, but none remained significant after false discovery rate correction from multiple comparisons. *CD163* gene was recently proposed as a potential mediator for small vessel injury in early Alzheimer disease.^44^ However, currently, no study has found a direct association between any of the seven biomarkers to the classic cSVD imaging markers, including WMH, lacunes, CMBs, and enlarged perivascular spaces. It is possible these represent true causal association that could be definitely demonstrated in a larger sample size but this needs testing in further studies.

This study has several strengths. To our knowledge, this is the first study to investigate whether CMBs are associated with a local inflammatory response or an increase in BBB leakage in vivo. In addition, our cohort consists of well-defined patients with sporadic cSVD. This approach minimizes the potential confounding effects from coexisting pathologies that could influence study outcomes.

However, it also has limitations to our study. First, as highlighted above, we cannot exclude transient increases in inflammation or BBB leakage associated with CMBs at their onset. Second, the number of CMB cases remained relatively small, and there were incomplete imaging data of several participants. Third, small CMBs cannot be visualized resulting in an inaccurate number of CMBs.^45^ Moreover, the hypointense lesion size on SWI may not reflect the true size of CMBs due to the blooming effect. Lastly, although 18 kDa TSPO PET is widely used as a reliable method to assess microglial activation, there are ongoing debates regarding whether it reflects true microglial activation or merely concentration.^46^ Therefore, PK11195 binding in our study may indicate the concentration of the microglial rather than their activation status.

In conclusion, using PET-MRI and systemic inflammation biomarkers, we did not find evidence that CMBs are independently associated with BBB leakage, microglial activation, or systemic inflammation in cSVD.

## Article Information

### Acknowledgments

The authors acknowledge the help of the radiographers at the Wolfson Brain Imaging Center, University of Cambridge, in scanning the subjects.

### Sources of Funding

Recruitment was supported by the National Institute for Health Research (NIHR) Clinical Research Network. Acquisition of the data was funded by a Medical Research Council experimental medicine grant (MR/N026896/1). L. Cai’s salary is funded by a joint grant from the British Heart Foundation and Dutch Heart Foundation (ref: SP/F/22/150028). Infrastructural support was provided by the Cambridge British Heart Foundation Center of Research Excellence (RE/24/130011) and the NIHR Cambridge Biomedical Research Center (NIHR203312). The views expressed in this publication are those of the authors and not necessarily those of the NIHR, National Institutes of Health (NHS), or UK Department of Health and Social Care.

### Disclosures

None.

### Supplemental Material

Tables S1–S3

Figure S1

STROBE Checklist
